# Direct cost of dengue hospitalization in Zhongshan, China: Associations with demographics, virus types and hospital accreditation

**DOI:** 10.1371/journal.pntd.0005784

**Published:** 2017-08-03

**Authors:** Jing Hua Zhang, Juan Yuan, Tao Wang

**Affiliations:** 1 School of Business, Macau University of Science and Technology, Taipa, Macau, China; 2 Zhongshan Center for Disease Control and Prevention, Zhongshan, China; University of Michigan, UNITED STATES

## Abstract

**Background:**

Zhongshan City of Guangdong Province (China) is a key provincial and national level area for dengue fever prevention and control. The aim of this study is to analyze how the direct hospitalization costs and the length of stay of dengue hospitalization cases vary according to associated factors such as the demographics, virus types and hospital accreditation.

**Method:**

This study is based on retrospective census data from the Chinese National Disease Surveillance Reporting System. Totally, the hospital administrative data of 1432 confirmed dengue inpatients during 2013–2014 was obtained. A quantile regression model was applied to analyze how the direct cost of Dengue hospitalization varies with the patient demographics and hospital accreditation across the data distribution. The Length of Stay (LOS) was also examined.

**Main findings:**

The average direct hospitalization cost of a dengue case in this study is US$ 499.64 during 2013, which corresponded to about 3.71% of the gross domestic product per capita in Zhongshan that year. The mean of the Length of Stay (LOS) is 7.2 days. The multivariate quantile regression results suggest that, after controlling potential compounding variables, the median hospitalization costs of male dengue patients were significantly higher than female ones by about US$ 18.23 (p<0.1). The hospitalization cost difference between the pediatric and the adult patients is estimated to be about US$ 75.25 at the median (p<0.01), but it increases sharply among the top 25 percentiles and reaches US$ 329 at the 90^th^ percentile (p<0.01). The difference between the senior (older than 64 years old) and the adult patients increases steadily across percentiles, especially sharply among the top quartiles too. The LOS of the city-level hospitals is significantly shorter than that in the township-level hospitals by one day at the median (p<0.05), but no significant differences in their hospitalization costs.

**Conclusions:**

The direct hospitalization costs of dengue cases vary widely according to the associated demographics factors, virus types and hospital accreditations. The findings in this study provide information for adopting hospitalization strategy, cost containment and patient allocation in dengue prevention and control. Also the results can be used as the cost-effective reference for future dengue vaccine adoption strategy in China.

## Author summary

There is little literature estimating dengue disease burdens and treatment costs worldwide; however, still fewer studies focus on the hospitalization cost of Dengue. Using the quantile regression method to analyze the administrative data of 1,432 confirmed dengue inpatients in Zhongshan City (Guangdong, China) during 2013 and 2014, this study examines the relationship of the direct cost of Dengue hospitalization and the associated factors. The Length of Stay (LOS) was also examined. The findings in this study will help to explain how the hospitalization cost varies with associated factors, providing information for adopting hospitalization strategy, cost containment and patient allocation in dengue prevention and control, as well as reference for future dengue vaccine cost-effective analysis.

## Introduction

Dengue fever (dengue) is an acute infectious disease caused by infection from any one of four serotypes of dengue virus (DENV1-4) transmitted by *Aedes* mosquitoes. Dengue virus infection in humans often shows no symptoms, but can cause a wide range of clinical manifestations, from mild fever to potentially fatal dengue shock syndrome [1]. It is estimated that about 3.6 billion people live in tropical and sub-tropical dengue-endemic areas and at least 50 million dengue infections occur annually.

Rapid, unorganized urbanization and a massive influx of migrants into these urban and suburban areas have resulted in this becoming a more urgent issue[2]. During recent decades, there has also been an increase in the rates of severe illness and hospitalizations of dengue cases [3,4]. Dengue has been a notifiable disease in China since 2005, essentially turning into a long-term threat in Southern China, when a serious epidemic broke out in Guangdong Province during 2014 [5][6] [7].

Dengue brings heavy socio-economic burdens, particularly given the limited public health resources and infrastructure in epidemic areas during outbreaks [2,4,8,9]. There is a limited amount of literature estimating dengue disease burdens and treatment costs worldwide [9,10], and even fewer studies focused on the hospitalization cost or the associated factors that account for the differences in the costs [11,12]. In-depth analysis in individual country settings is also needed due to significant diversities in patient demographics, treatment sites and country settings [13]. Currently, there is no study about the economic cost of dengue fever in China, or specifically about the hospitalization cost of dengue cases in China.

Zhongshan City of Guangdong Province (China) was the first city with autochthonous dengue cases reported in mainland China [14]. It experienced outbreaks of dengue during 2013 to 2014, with a total of 1,432 cases reported. The prevention and control of dengue has been given high priority in Zhongshan. Considering the high level of inapparent-to-symptomatic (I:S) ratio and virus transmission risks in Zhongshan [14], the CDC of Zhongshan required all diagnosed dengue cases, including non-fatal cases, to be hospitalized in the quarantine wards of local hospitals.

The aim of this study is to analyze how the direct costs of the hospitalization and the length of stay (LOS) vary with the patient demographics and hospital accreditations. Direct costs (or financial costs) represent the actual expenditure on health care services and goods, providing direct information about monetary amounts needed to be paid for the resources; therefore it is recommended from a practical standpoint [15]. The analysis was conducted from the public health payer’s perspective. The findings are expected to provide cost information for adopting hospitalization strategy, as well as cost containment and patient allocation in dengue prevention and control. The hospitalization cost information can also be used as cost-effective reference for future dengue vaccine adoption strategy in China.

## Methods

### Ethics statement

The study methods were reviewed and approved by research review boards at the Macao University of Science and Technology as well as the Zhongshan Center for Disease Control and Prevention (Zhongshan CDC). An anonymized dataset of all confirmed dengue inpatients was obtained from the Chinese National Disease Surveillance Reporting System (CNDSRS). Patient names and ID number information were not exported from the CNDSRS database.

### Study location

**Fig 1:**
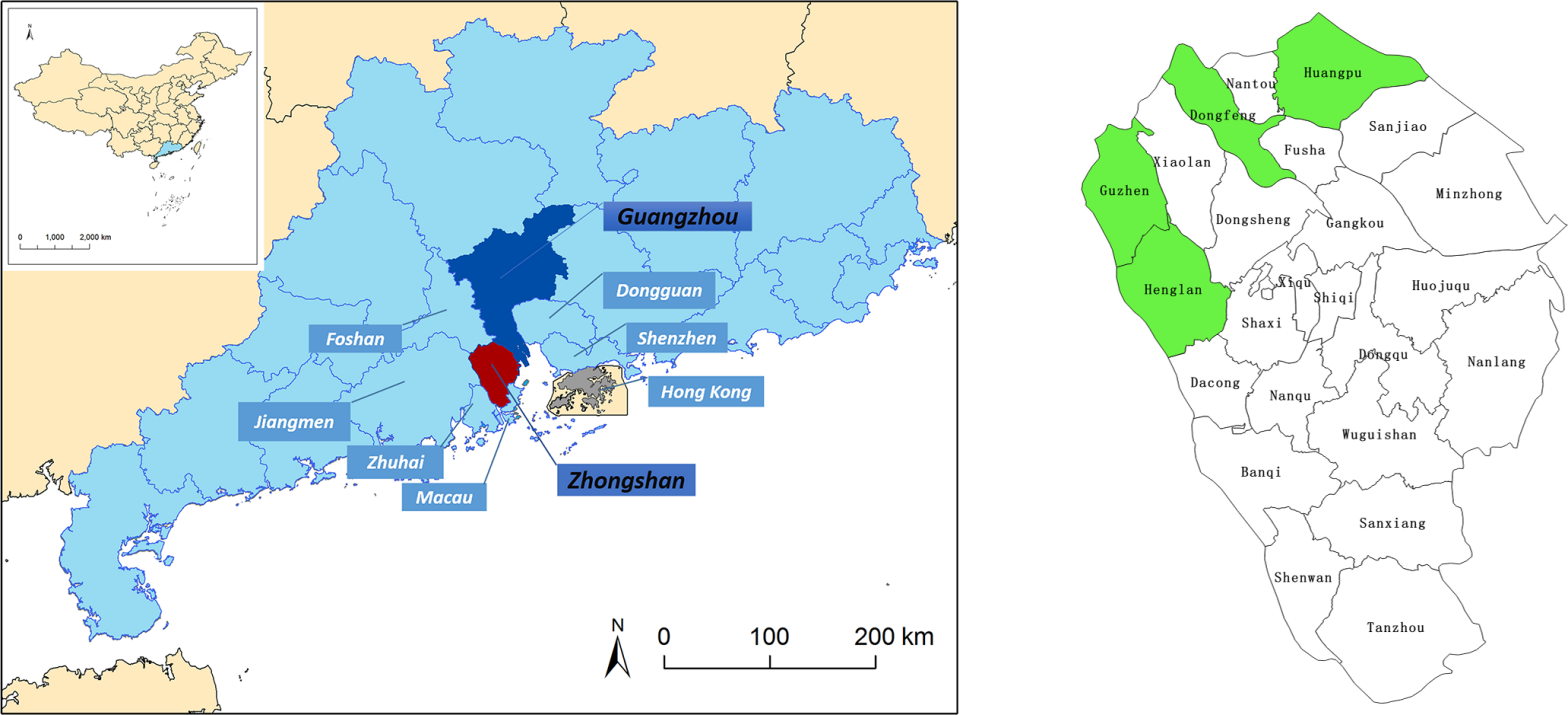
The location of Zhongshan City Guangdong Province, China, and a map of the townships in Zhongshan City. This figure on the left is adapted from Fig 1 from Zhang et al pntd.0004473 [27].

Zhongshan City covered an area of 1,800 sq km with a total population of about 3.2 million in 2014. Located along the west side of the Pearl River Delta, Zhongshan City is in close vicinity to other major international cities in southern China, such as Guangzhou, Hong Kong and Macao (Fig 1). With its warm and humid subtropical climate, Zhongshan has become the ideal breeding place of *Aedes* [14,16,17]. The primary vector during the studied dengue epidemic is *Aedes albopictus*, which is also the locally predominant mosquito species [5,14,18]. Virus Type I was the predominant serotype in circulation through the years studied [19].

### Healthcare system background

The healthcare system in China is a largely public hospital based delivery system under the administration of the National Health and Family Planning Commission (NHFPC) of China. A national hospital accreditation system classifies hospitals into primary, secondary and tertiary levels according to characteristics such as numbers of beds, professional criteria of healthcare teams, diagnosis and treatment equipment possessions and operation area sizes[20]. Always located in major cities, tertiary hospitals are at the highest level and with characteristics such as more than 500 patient beds, leading healthcare specialists, as well as advanced diagnostic and treatment equipments. They also carry out medical education and research. A secondary hospital has 100–499 beds and a primary one has 20–99 beds.

There are strict health care pricing regulations in China. During year 2013–2014 in Guangdong Province, the basic medical service prices in tertiary hospitals were set to be about 20% higher than in the secondary hospitals, in which the service price level was about 10–20% higher than those of primary hospitals [21,22]. The prices of prescription drug and diagnostic tests were set to be the same across hospitals. Under this distorted pricing system, during 2012–2014 in Guangdong Province healthcare service fees only accounted for about 10% of the total health care expenditure, whereas prescription drug account for more than 37%, while diagnostic test fees and treatment fees together account for about 40% [23,24].

China has not yet implemented a strict health care referral and gate-keeping system; patients are free to self-refer to any hospitals. Although the national health insurance program provides a higher reimbursement rate for local primary hospitals, most patients in China have strong preferences for secondary and tertiary hospitals, regardless of the severity of their sickness [20]. Consequently, tertiary hospitals always face very high patient demands, while primary hospitals have a much lower demand.

By 2013, the public health insurance coverage for all Zhongshan residents had reached 98.8% [25]. 80–90% of the hospitalization costs of dengue patients were covered by public health insurance. For dengue patients who didn’t have any form of health insurance, the Zhongshan City government provided subsidies to cover up to 90% of the hospitalization expenses.

### Hospitals’ profiles

Zhongshan City has two tertiary hospitals and four secondary hospitals, which together are called city-level hospitals. Since all primary hospitals in Zhongshan are located in townships, they are also called township-level hospitals. There are in total 6 city-level hospitals and 23 township-level hospitals in Zhongshan designated to admit dengue patients [26]. Except for one not-for-profit city-level hospital, all other hospitals were public ones operated by local governments.

### Data collection

As a notifiable infectious disease, individual dengue cases, including clinically diagnosed and laboratory confirmed cases, are required to be reported through the CNDSRS within 24 hours after diagnosis [27]. Dengue case data in Zhongshan City during the period of 1^st^ January 2013 to 31^st^ December 2014 were collected from the official database of CNDSRS. All confirmed cases are included in this study without exclusion, because all dengue cases during the epidemic were required to be hospitalized by Zhongshan CDC.

The dengue cases were diagnosed based on standardized laboratory tests and clinical investigations according to Guangdong Dengue Guidelines for Diagnosis and Treatment (2013), which reflected the updating in the Guidelines of WHO 2009 [28,29]. Dengue cases were confirmed either by Dengue NS1 antigen ELISA detection, or the IgM/IgG capture ELISA kits from the serum samples of the suspected cases [30].

Patients were to be hospitalized in the first hospital where they were diagnosed, unless that hospital was over-occupied. Dengue with warning signs and severe dengue cases as defined by the Guidelines of WHO 2009 [28,29] were required to be transferred to the city-level hospitals in Zhongshan. The Dengue treatment in hospitals in Zhongshan followed the national guidelines and Guangdong Dengue Guidelines for Diagnosis and Treatment (2013), which is based on the Guidelines of WHO (2009) [28,29].

The direct cost of hospitalization was based on the patient records maintained by hospitals. It was the aggregated amount of all expenses occurred during the hospitalization and included medical services, medication supplies, laboratory tests, X-rays, boarding, ICU fee and other special procedures if required. This variable was not itemized and the data system contained no numerical information about the sub-items. Since the hospital treatments followed WHO Guidelines (2009) [28,29], it can be inferred that hospital personnel cost (salaries and allowances) accounted for about half of the total hospitalization cost for dengue cases [12,13]. The cost was reported in local currency (RMB), and later converted to United States Dollars (US$) according to the average exchange rate for 2013–2014, 1 USD = 6.2 RMB. Using the inflation index of Zhongshan, we deflated the medical costs to 2013, the base year.

Per capita income data aggregated on township level were obtained from the Zhongshan Year Book (2013–2014) and linked to individual entries.

### Statistical methods

#### Quantile regression model

In this study, we adopted the quantile regression method to analyze how the hospitalization costs and LOS at high or low quantile of the distribution vary according to the patient demographics, virus types and hospital accreditation. The quantile regression parameter estimates the change at a specified quantile of the conditional distribution of the dependent variable associated with one unit change in the explanatory variable [31]. This model makes no assumption about the distribution of the underlying data and provides unbiased estimates even with the lack of normal distribution or the presence of outlier observations [31]. Since healthcare utilization data are usually highly skewed [32] and with unequal variance across the distribution of sample group responses[33,34], the quantile regression approach can be a good alternative to Ordinary Least Squared (OLS) regression. Also, a comprehensive picture of the effects of the associated factors can be obtained through quantile regression [31]. Due to these merits, the quantile regression method has been widely applied in healthcare cost analysis [35–38].

We used the command ***bsqreg*** [39] in Stata 12 statistical package (Stata Corp LP, College Station, TX, USA) to apply the analysis. Standard errors were determined using a bootstrapping method with 1000 repetitions [40,41]. OLS regressions were also performed to compare the results. Additionally, we performed inter-quantile tests using the command *iqreg* in Stata 12 to check whether differences across the quantiles are significant.

#### Variables

Each entry of the case data contains case ID, patient age, gender, occupation, township ID, dengue virus type, total direct cost of hospitalization, length of stay, the name of and the admission hospital ID.

The response variables examined in this study refers to the total direct cost (in 2013 US dollars) or the length of stay (LOS) of a dengue hospitalization episode during the epidemic of 2013–2014. The explanatory variables of healthcare utilization include patient gender, age group, virus type, hospital accreditation type, year of hospitalization, high risk area indicator, an interaction term of the high risk area and the year, as well as per capita income aggregated on a township level.

Patients are classified into three groups based on their ages. The base group is the Adult Group (15–64 years). The Pediatric Group refers to those who are younger than 15 years old (0–14 years), and the Senior Group includes those who are 65 years and older (65> =).

There were only dengue Virus Type I, Type II and Type III confirmed in Zhongshan. Hospital accreditation type has township and city level as discussed in Healthcare System Background section.

Residential areas of the patients are classified into High and Low Risk areas according to the patient’s township. The four townships of Zhongshan City (Huangpu, Guzhen, Dongfeng and Henglan), which had an outbreak in 2013, are classified as High Risk Areas. The rest of the townships were regarded as Low Risk Areas. A dummy variable is included to identify cases reported in year 2014. We also include an interaction term for year 2014 and High Risk Area to capture the variation of the utilization among the cases from High-Risk-Areas in 2014.

## Results

### Descriptive statistics

A total of 1,432 confirmed dengue cases in Zhongshan, 809 cases in 2013 and 623 cases in 2014, were reported during the epidemic period of 2013 to 2014. There were no life-threatening cases in the epidemic periods, however, patient severity at admission was not included in the database.

Table 1 shows the demographic and epidemic characteristics of the hospitalized dengue patients in Zhongshan City during 2013–2014. The incidence rates were highest among the senior group (65 > =), while lowest among the pediatric Group (0–14 years old) in both year 2013 and 2014. In 2013 the city-level hospitals admitted approximately 86% of all dengue patients. Because the sudden epidemic outbreak concentrated in four townships, the local township-level hospitals were fully occupied and had to transfer the rest of the patients to city-level hospitals.

**Table 1:** Demographic and epidemic characteristics of hospitalized dengue cases in Zhongshan City(China) during 2013–2014.

	2013			2014			Total cases			Chi2
	N	%	Incidence (per 100,000)	N	%	Incidence (per 100,000)	N	%	Incidence (per 100,000)	
**Gender**										
female	403	49.94	25.23	280	44.94	17.40	683	47.77	21.33	0.067
male	406	50.06	25.76	343	55.06	21.66	749	52.23	23.67	
**Age group**										
Pediatric (0–14)	69	8.53	18.64	34	5.46	9.13	103	7.19	13.87	0.003
Adult (15–64)	678	83.81	25.44	542	87.00	20.22	1,220	85.20	22.82	0.023
Senior (65> =)	62	7.66	44.70	47	7.54	33.69	109	7.61	39.18	
**Virus type**										
I	336	41.53		113	18.14		449	31.35		
II	395	48.83		441	70.79		836	58.38		
III	78	9.64		69	11		147	10.27		< .0001
**Hospital accreditation**										
Township level	111	13.72		301	48.31		412	28.77		
City level	698	86.28		322	51.69		1020	71.23		< .0001
**Epidemic area**										
Low risk areas	271	33.50		547	87.80		818	57.12		
High risk areas	538	66.50		76	12.20		614	42.88		< .0001
**Total**	**809**	**100**	**25.49**	**623**	**100**	**19.51**	**1,432**	**100**	**22.49**	

**Fig 2:**
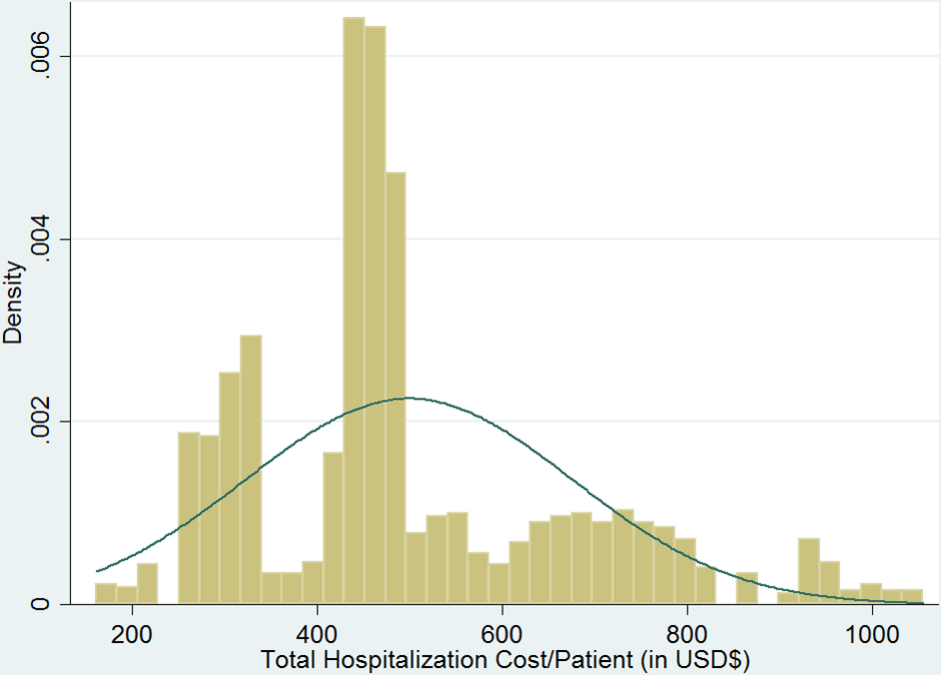
Distribution of the total hospitalization cost per patient in Zhongshan City during 2013–2014 (in 2013 USD $).

Table 2 reports the descriptive statistics of total hospitalization cost and LOS of all Dengue patients in Zhongshan during 2013–2014 (all costs are in 2013 US$). The average hospitalization cost of a dengue case is US$ 499.64. Despite the lower medical service price level and usually lower disease severity in township-level hospitals, their hospitalization costs were US$ 537.86 on average, higher than those in city-level hospitals by about US$50.00. Fig 2 shows the distribution of the Dengue hospitalization cost. The cost data is highly skewed to the right due to the very high treatment cost of the top ten percent of patients. The LOS is about seven days on average, the same in both groups of hospitals.

**Table 2:** Descriptive statistics of total hospitalization cost, and length of stay of all dengue patients in Zhongshan City during 2013–2014.

	Total cost of hospitalization per case (in 2013 US$)					Length of Stay (LOS)				
	Mean	SD	Ttest (p-value)	Median	IQR	Mean	SD	Ttest (p-value)	Median	IQR
**Year**										
2013	439.65	156.92		447.26	162.26	7.2	2.3		7	1
2014	577.54	171.47	< .0001	557.21	263.01	7.2	2.2	0.781	7	2
**Gender**										
Female	498.22	178.29		464.19	203.45	7.2	2.3		7	2
Male	500.94	176.09	0.77	462.17	176.22	7.1	2.3	0.410	7	1
**Age (years)**					0.00					
Pediatric (0–14)	588.29	180.07		533.06	264.59	8.9	3.0		7	4
Adult (15–64)	465.70	143.70	< .0001	455.36	166.45	6.6	1.4	< .0001	7	1
Senior (65> =)	782.27	220.54	< .0001	863.23	304.32	11.7	3.2	< .0001	13	7
**Virus type**										
I	398.34	122.05		420.59	153.39	6.2	1.8		6	2
II	515.59	154.34	< .0001	473.55	198.18	7.1	1.8	< .0001	7	1
III	718.35	210.34	< .0001	734.58	391.77	10.5	3.0	< .0001	10	5
**Hospital** **accreditation**										
Township level	537.86	179.31		480.32	256.99	7.1	2.2			0
City level	484.20	173.90	< .0001	459.68	177.09	7.2	2.3	0.499	7	1
**Epidemic area**										
Low risk areas	521.20	184.28		470.25	243.54	7.0	2.2		7	1
High risk areas	470.90	162.78	< .0001	455.24	154.35	7.4	2.4	0.003	7	1
**Full sample**	499.64	177.09		463.71	178.70	7.2	2.3		7	1

Note: N = 1432

### Quantile regression results

#### Total cost of hospitalization per episode

**Fig 3:**
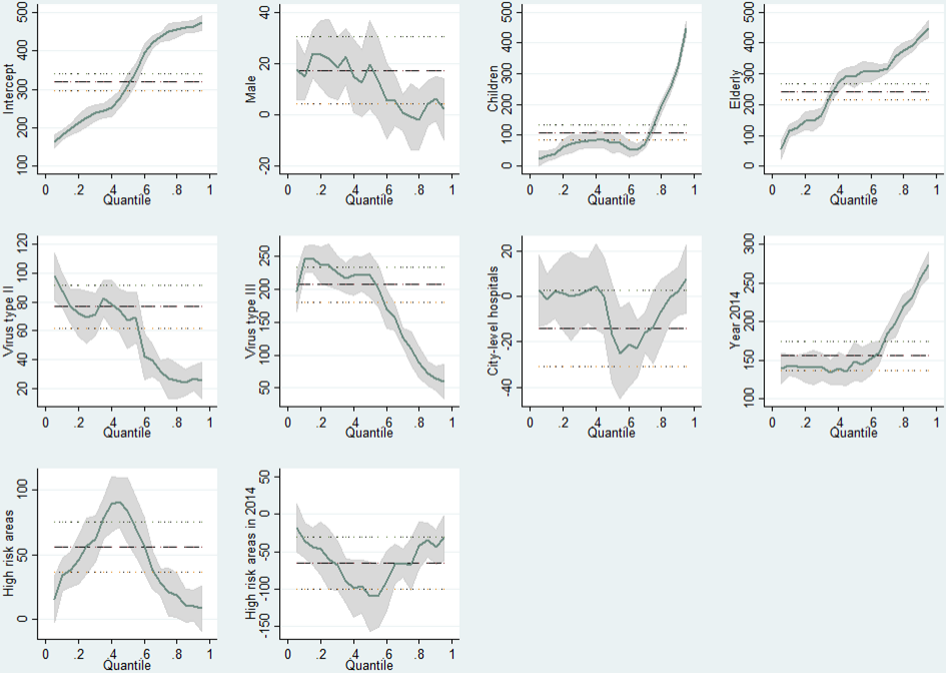
OLS and quantile regression results of the factors associated with the total hospitalization cost of dengue cases at Zhongshan City (China) (2013–2014).

The results in Table 3 shows that, *ceteris paribus*, the total hospitalization cost of a male dengue patient case is higher than that of a female patient case by US$17.14 at the mean (significant at the 5% level) and US$18.23 at the median (significant at the 10% level). This effect is especially significant among the lower quantiles with the highest difference of US$22.21 at the 25th percentile, but not at the 75th percentile. Except for the variable City-level hospital, the coefficients of other tested variables, such as Virus Types, Year 2014, High risk areas and the interaction term are all statistically significant across the quantiles. The costs of Virus II and III are consistently higher than that of Virus I by about US$76.99 and US$206.66 on average respectively (p<0.01). Fig 3 has clearly demonstrated the patterns of the changes in the coefficients across the quantiles. Table 4 displays the results of interquartile tests, which are consistent with the results reported in Table 3.

**Table 3:** Quantile regression results of the total hospitalization cost of dengue cases at Zhongshan (China) (2013–2014).

Variables	OLS	10th Percentile ^a^	25th Percentile	50th Percentile	75th Percentile	90th Percentile
Male	17.138** ^b^	17.639***	22.211***	18.226*	0.161	8.857*
	(6.698)	(6.021)	(7.674)	(9.488)	(6.046)	(5.237)
**Age group** ^**c**^						
Adult (15–64)	ref.					
Pediatric (0–14)	108.885***	39.518**	71.068***	75.249***	127.776**	329.239***
	(17.302)	(18.470)	(23.619)	(15.667)	(50.317)	(75.595)
Senior (65> =)	242.451***	117.522***	150.184***	292.802***	355.087***	416.789***
	(21.114)	(36.574)	(29.282)	(32.966)	(29.263)	(17.482)
**Virus Type**						
Virus Type I	ref.					
Virus Type II	76.985***	83.651***	68.969***	69.706***	27.097***	28.871***
	(7.444)	(7.638)	(12.120)	(22.671)	(6.626)	(6.462)
Virus Type III	206.658***	243.074***	235.776***	222.198***	111.526***	59.839***
	(14.041)	(25.689)	(13.540)	(25.051)	(20.478)	(8.041)
**Hospital accreditation**						
Township level	ref.					
City level	-14.943*	-2.683	-0.958	-20.055	-13.601	7.304
	(8.999)	(6.021)	(7.898)	(19.236)	(11.115)	(7.142)
Year 2014	151.633***	133.538***	136.535***	143.020***	194.935***	261.503***
	(12.521)	(9.523)	(11.218)	(24.660)	(16.268)	(11.017)
High risk areas	55.972***	35.371***	55.774***	85.678***	20.706***	10.335**
	(9.082)	(8.469)	(14.880)	(26.610)	(7.283)	(4.800)
High risk areas * Year2014	-66.665***	-36.630***	-59.147***	-115.940***	-61.705	-41.339*
	(17.723)	(12.940)	(18.093)	(37.613)	(38.984)	(21.348)
Constant	290.633***	120.908***	208.843***	264.165***	441.043***	509.126***
	(47.290)	(40.071)	(48.010)	(90.193)	(50.786)	(36.945)
Observations	1,432	1,432	1,432	1,432	1,432	1,432
R-squared	0.507					

**Note:** a. The percentiles of the total hospitalization cost of all Dengue cases in the dataset.

b. Robust standard errors in parentheses.

c. Per Capita Income variable was included but insignificant.

* p<0.1

** p<0.05

*** p<0.01

**Table 4:** Interquantile regression results of the total hospitalization cost of dengue cases at Zhongshan City (China) (2013–2014).

Variables	.10-.25 ^a^	.25-.50	.10-.50	.50-.75	.75-.90	.50-.90
Male	7.290	-2.429	4.861	-20.323**	6.966	-13.356
	(7.083)^b^	(8.241)	(9.557)	(8.175)	(5.818)	(9.129)
**Age group** ^**c**^						
Adult (15–64)	ref.					
Pediatric (0–14)	39.723**	2.191	41.914*	53.616	194.194***	247.809***
	(19.456)	(21.208)	(21.797)	(45.455)	(63.635)	(73.679)
Senior (65> =)	32.084	140.800***	172.884***	65.481**	65.388**	130.869***
	(34.474)	(32.969)	(42.543)	(28.798)	(25.686)	(31.615)
**Virus Type**						
Virus Type I	ref.					
Virus Type II	-18.256	-1.868	-20.125	-40.551**	0.326	-40.225*
	(11.206)	(21.768)	(21.489)	(20.492)	(6.247)	(21.392)
Virus Type III	-9.398	-14.714	-24.112	-110.135***	-46.966***	-157.101***
	(23.561)	(21.882)	(31.888)	(22.060)	(17.976)	(24.356)
**Hospital accreditation**						
Township level	ref.					
City-level hospital	1.613	-16.774	-15.161	2.903	15.906*	18.809
	(6.592)	(16.102)	(17.879)	(16.127)	(9.159)	(17.614)
Year 2014	-3.338	7.968	4.629	49.120**	59.610***	108.730***
	(8.047)	(19.116)	(22.246)	(20.242)	(10.902)	(22.562)
High risk areas	22.097*	27.352	49.449*	-62.997***	-10.353	-73.351***
	(13.043)	(26.765)	(25.314)	(24.025)	(6.840)	(25.556)
High risk areas* Year 2014	-24.843	-50.021	-74.864**	42.657	23.553	66.210*
	(15.635)	(32.439)	(34.189)	(40.602)	(34.321)	(37.014)
Constant	44.699***	81.300***	125.998***	140.968***	13.933	154.901***
	(11.773)	(18.682)	(20.692)	(17.513)	(8.763)	(19.768)
Observations	1,432	1,432	1,432	1,432	1,432	1,432

**Note:** a. The percentiles of the total hospitalization cost of all Dengue cases in the dataset.

b. Robust standard errors in parentheses.

c. Per Capita Income variable was included but insignificant.

* p<0.1

** p<0.05

*** p<0.01

#### Length of stay

Using the same quantile regression method, we examined the Length of Stay. The results are reported in Table 5. The OLS results in the first row of Table 5 show that the length of stay among the male patients is significantly longer than the female patients by about 0.2 days on average. However, the results of the quantile regression on Table 5 show that there is no significant difference across the quantiles. The coefficients of LOS for Virus Type II and III are still systematically higher than those of Virus Type I.

**Table 5:** Quantile regression results of the length of stay of dengue cases at Zhongshan City (China) (2013–2014).

	OLS	10th Percentile ^a^	25th Percentile	50th Percentile	75th Percentile	90th Percentile
**Male**	0.202**^b^	0.000	0.000	0.000	0.000***	0.000
	(0.085)	(0.209)	(0.022)	(0.038)	(0.000)	(0.117)
**Age group ^c^**						
Adult (15–64)	ref.					
Pediatric (0–14)	1.788***	0.000	1.000***	1.000***	2.000***	5.000***
	(0.257)	(0.254)	(0.383)	(0.214)	(0.745)	(0.963)
Senior (65> =)	3.982***	1.000**	2.000***	5.000***	6.000***	7.000***
	(0.326)	(0.436)	(0.683)	(0.605)	(0.236)	(0.337)
**Virus Type**						
Virus Type I	ref.					
Virus Type II	0.761***	1.000***	1.000***	1.000**	0.000	1.000***
	(0.091)	(0.000)	(0.022)	(0.433)	(0.200)	(0.122)
Virus Type III	2.831***	3.000***	3.000***	3.000***	2.000***	2.000***
	(0.209)	(0.423)	(0.189)	(0.477)	(0.278)	(0.351)
**Hospital accreditation**						
Township level	ref.					
City level	-0.205*	0.000	0.000	-1.000**	-0.000**	-0.000
	(0.105)	(0.022)	(0.000)	(0.437)	(0.000)	(0.164)
Year 2014	0.312**	1.000***	1.000**	0.000	-0.000	1.000**
	(0.129)	(0.199)	(0.478)	(0.433)	(0.059)	(0.505)
High risk areas	0.742***	1.000***	1.000*	1.000***	-0.000	-0.000
	(0.123)	(0.369)	(0.587)	(0.352)	(0.191)	(0.114)
High risk areas * Year 2014	-0.680***	-1.000***	-1.000*	-1.000*	0.000	0.000
	(0.224)	(0.373)	(0.587)	(0.510)	(0.292)	(1.122)
Constant	5.616***	4.000***	4.000***	6.000***	7.000***	7.000***
	(0.138)	(0.066)	(0.478)	(0.471)	(0.191)	(0.160)
Observations	1,432	1,432	1,432	1,432	1,432	1,432
R-squared	0.509					

**Note:** a. The percentiles of the total hospitalization cost of all Dengue cases in the dataset.

b. Robust standard errors in parentheses

c. Per Capita Income variable was included but insignificant.

* p<0.1

** p<0.05

*** p<0.01

In city-level hospitals, the LOS is significantly shorter (0.2 days on average and one day at the median) than that of the township-level. The coefficients of both Year 2014 and high risk townships indicators are significant on average, as well as on lowest percentiles.

The interaction term of High Risk Areas* for Year 2014 indicates that in the dengue epidemic year of 2014, the increase of LOS among the high risk townships on average is less than those among the low risk townships. This effect was especially significant on the lowest quantile and the median.

## Discussion

The average hospitalization cost of a dengue case in this study was US$499.64 during 2013, which corresponded to 3.71% of the gross domestic product per capita in Zhongshan that year. Both the hospitalization cost and LOS were very close to those of private hospitals in Brazil in 2010, which were US$515.80 and 7.3 days respectively [11]. However, the hospitalization cost accounted for only 2.5% of the GDP per capita of Dourados, Brazil in 2010 [11].

The gender difference in the hospitalization cost may be due to the differences in disease awareness and health-care seeking behaviors. Males often have lower knowledge scores and a lower perception of susceptibility to dengue [42,43]. In the case of being infected by dengue, males are more likely than women to delay health-care seeking behaviors [44,45]. As the results, male patients may have had greater severity when they were hospitalized. Additionally, the impact of different physiologies can not be ruled out [46].

The pediatric and the senior patients consistently have greater total costs and longer LOSs than the adult patients. These results are consistent with existing literature [11–13,47]. Among the pediatric cases, only the top quarter has higher total cost than the mean. However, the total costs of the senior cases increased steadily across quartiles, and sharply at the top quartiles.

The higher costs for Virus Type II and III cases are consistent with the fact that these patients usually have more serious symptoms, especially Virus III. The cost differences between Virus Type I and the other two were as high as US$87.00 to US$244.90 at the 10^th^ percentile (p<0.1), but decreased to US$27.00 and US$63.70 at the 90^th^ percentile (p<0.1). We are not aware of any study comparing the hospitalization costs of dengue patients based on the types of dengue virus, though there are some reports based on the types of care, for example, general ward and ICU [11–13,47].

Both the total hospitalization cost and LOS in the city-level hospitals tended to be shorter on average than in the township-level hospitals, *ceteris paribus*, though the effects are not significant across the quartiles. During 2013–2014, the hospital bed density in Zhongshan was 3.98 beds/1,000 people on average, which was slightly higher than the average level in Guangdong Province [26]. Tertiary hospitals are always in high demand by patients. The bed occupancy rate of tertiary hospitals in Guangdong Province was 97.6% on average, whereas the township-level hospitals had only 55% occupancy [24]. Hospitals may shorten the LOS when facing high demand pressures and patient volume[48,49], During the epidemic time, the demand for tertiary hospitals is even much higher than their capacities [11].

At last, the higher level of hospital utilization in year 2014 and in the high risk areas is likely due to the intensified dengue prevention and control activities [12]. In the year 2014, the hospital utilizations in the high risk areas had smaller increases than those in low risk areas. This may be due to the already intensified treatment among the high risk area in 2013, therefore it did not increase as much as in the low risk area in 2014. Meanwhile, it is also possible that some residents from high risk epidemic areas might have obtained enhanced immunity in 2014 due to inapparent dengue infection of the same type virus during the outbreak of 2013 [19,30].

Considering socioeconomic factors have various impacts on preventive and health care seeking behaviors in China [50], we performed analysis using occupation as a control variable of socioeconomic factors but found the estimated coefficients insignificant. Considering that internal migrant workers may not have equal access to local housing, health care, and social services [51], we tested with an indicator of local citizenship status but again found no significant results.

### Limitations

The epidemiological characteristics vary largely during outbreaks in different regions. Caution is necessary when generalizing the findings about costs of dengue hospitalization found in this study. The findings are also limited by data availability. First, the database does not contain the host susceptibility or its immune status, which usually mean large differences in the severity of the infections. Second, we could not further analyze the composition of hospitalization costs, because the subcategory data, such as laboratory diagnosis cost, drug cost, medical personnel cost or boarding costs, is not available in the database. Third, no detailed individual level socioeconomic information is available from the database, even though these factors affect the risks of dengue infections through the environmental mechanisms of water and sanitation, housing and work conditions etc.[4,52].

In the future, if the disease surveillance system of China could be improved to collect more detailed disease-specific information, it could generate even more enriched information to enable efficient policy making for disease prevention and control.

## References

[1] Guzman MG, Harris E. Dengue. The Lancet. 2015;385: 453–465. 10.1016/S0140-6736(14)60572-9 25230594

[2] Bhatt S, Gething PW, Brady OJ, Messina JP, Farlow AW, Moyes CL, et al The global distribution and burden of dengue. Nature. 2013;496: 504–507. doi: 10.1038/nature12060 23563266 10.1038/nature12060 PMC3651993

[3] Gubler DJ. Epidemic dengue/dengue hemorrhagic fever as a public health, social and economic problem in the 21st century. Trends Microbiol. 2002;10: 100–103. 11827812 10.1016/s0966-842x(01)02288-0

[4] Gubler DJ. Dengue, Urbanization and Globalization: The Unholy Trinity of the 21(st) Century. Trop Med Health. 2011;39: 3–11. 10.2149/tmh.2011-S05 PMC3317603 22500131

[5] Health Department of Guangdong Province, China Guangdong Province dengue epidemic events report 12 15 2014.

[6] Chen B, Liu Q. Dengue fever in China. The Lancet. 2015;385: 1621–1622. 10.1016/S0140-6736(15)60793-0 25943817

[7] Wu JY, Lun ZR, James AA, Chen XG. Dengue Fever in mainland China. Am J Trop Med Hyg. 2010;83: 664–671. doi: 10.4269/ajtmh.2010.09-0755 20810836 10.4269/ajtmh.2010.09-0755 PMC2929067

[8] Constenla D, Garcia C, Lefcourt N. Assessing the Economics of Dengue: Results from a Systematic Review of the Literature and Expert Survey. Pharmacoeconomics. 2015;33: 1107–1135. doi: 10.1007/s40273-015-0294-7 26048354 10.1007/s40273-015-0294-7

[9] Shepard DS, Undurraga EA, Halasa YA, Stanaway JD. The global economic burden of dengue: a systematic analysis. The Lancet Infectious Diseases. 2016. 10.1016/S1473-3099(16)00146-8 27091092

[10] Beatty ME, Beutels P, Meltzer MI, Shepard DS, Hombach J, Hutubessy R, et al Health economics of dengue: a systematic literature review and expert panel's assessment. Am J Trop Med Hyg. 2011;84: 473–488. doi: 10.4269/ajtmh.2011.10-0521 21363989 10.4269/ajtmh.2011.10-0521 PMC3042827

[11] Machado AAV, Estevan AO, Sales A, da Silva Brabes, Kelly Cristina, Croda J, Negrão FJ. Direct costs of dengue hospitalization in Brazil: public and private health care systems and use of WHO guidelines. PLoS Negl Trop Dis. 2014;8: e3104 doi: 10.1371/journal.pntd.0003104 25188295 10.1371/journal.pntd.0003104 PMC4154670

[12] Thalagala N, Tissera H, Palihawadana P, Amarasinghe A, Ambagahawita A, Wilder-Smith A, et al Costs of Dengue Control Activities and Hospitalizations in the Public Health Sector during an Epidemic Year in Urban Sri Lanka. PLoS Negl Trop Dis. 2016;10: e0004466 doi: 10.1371/journal.pntd.0004466 26910907 10.1371/journal.pntd.0004466 PMC4766086

[13] Suaya JA, Shepard DS, Siqueira JB, Martelli CT, Lum LC, Tan LH, et al Cost of dengue cases in eight countries in the Americas and Asia: a prospective study. Am J Trop Med Hyg. 2009;80: 846–855. 19407136

[14] Wang T, Wang M, Shu B, Chen XQ, Luo L, Wang JY, et al Evaluation of inapparent dengue infections during an outbreak in Southern China. PLoS Negl Trop Dis. 2015;9: e0003677 doi: 10.1371/journal.pntd.0003677 25826297 10.1371/journal.pntd.0003677 PMC4380470

[15] Constenla D, Garcia C, Lefcourt N. Assessing the economics of dengue: results from a systematic review of the literature and expert survey. Pharmacoeconomics. 2015;33: 1107–1135. doi: 10.1007/s40273-015-0294-7 26048354 10.1007/s40273-015-0294-7

[16] Fan J, Lin H, Wang C, Bai L, Yang S, Chu C, et al Identifying the high-risk areas and associated meteorological factors of dengue transmission in Guangdong Province, China from 2005 to 2011. Epidemiol Infect. 2014;142: 634–643. doi: 10.1017/S0950268813001519 23823182 10.1017/S0950268813001519 PMC9161228

[17] Qi X, Wang Y, Li Y, Meng Y, Chen Q, Ma J, et al The Effects of Socioeconomic and Environmental Factors on the Incidence of Dengue Fever in the Pearl River Delta, China, 2013. PLoS Negl Trop Dis. 2015;9: e0004159 doi: 10.1371/journal.pntd.0004159 26506616 10.1371/journal.pntd.0004159 PMC4624777

[18] CHEN QQ, MENG YJ, Yue L, QI XP. Frequency, Duration and Intensity of Dengue Fever Epidemic Risk in Townships in Pearl River Delta and Yunnan in China, 2013. Biomedical and Environmental Sciences. 2015;28: 388–395. doi: 10.3967/bes2015.055 26055568 10.3967/bes2015.055

[19] Guo R, Lin J, Li L, Ke C, He J, Zhong H, et al The prevalence and endemic nature of dengue infections in guangdong, South china: an epidemiological, serological, and etiological study from 2005–2011. PloS one. 2014;9. 10.1371/journal.pone.0085596 PMC3900419 24465613

[20] Eggleston K. Health care for 1.3 billion: An overview of China’s health system. Stanford Asia Health Policy Program Working Paper No 28. 2012.

[21] Gu S. Comparing the Healthcare Service Price in China: a Cross Regions Study., Fudan University (China), Dissertation. 2013.

[22] Liu X, Liu Y, Chen N. The Chinese experience of hospital price regulation. Health Policy Plan. 2000;15: 157–163. 10837038 10.1093/heapol/15.2.157

[23] WENG Y, ZOU L, CHEN Y. Study on Straightening out the Price Svstem of Medical Services in Guangdong. Chinese Health Economics. 2017;36: 70–72.

[24] Huang F. Healthcare reform in Guangdong (China) and the outlook. 2015;ST.

[25] ZMG ZMG. Zhongshang City Facts—Health care development. Dec. 2016.

[26] Zhou X, Wu W. Zhongshan Yearbook: Zhongshan City Bureau of Statistics; 2015.

[27] Zhang Y, Wang T, Liu K, Xia Y, Lu Y, Jing Q, et al Developing a Time Series Predictive Model for Dengue in Zhongshan, China Based on Weather and Guangzhou Dengue Surveillance Data. PLoS Negl Trop Dis. 2016;10: e0004473 doi: 10.1371/journal.pntd.0004473 26894570 10.1371/journal.pntd.0004473 PMC4764515

[28] WHO. Dengue Guidelines for Diagnosis, Treatment, Prevention And Control. 2009. 23762963

[29] National Health and Family Planning Commission (Guangdong, China). Guangdong Dengue Guidelines for Diagnosis and Treatment (2013). 2013: In press.

[30] Lin YP, Luo Y, Chen Y, Lamers MM, Zhou Q, Yang XH, et al Clinical and epidemiological features of the 2014 large-scale dengue outbreak in Guangzhou city, China. BMC infectious diseases. 2016;16: 102 doi: 10.1186/s12879-016-1379-4 26932451 10.1186/s12879-016-1379-4 PMC4774186

[31] Koenker R, Hallock K. Quantile regression: An introduction. Journal of Economic Perspectives. 2001;15: 43–56.

[32] Martelli CMT, Junior JBS, Parente, Mirian Perpetua Palha Dias, Zara, Amancio Ana Laura de Sene, Oliveira CS, Braga C, et al Economic Impact of Dengue: Multicenter Study across Four Brazilian Regions. PLoS Negl Trop Dis. 2015;9: e0004042 doi: 10.1371/journal.pntd.0004042 26402905 10.1371/journal.pntd.0004042 PMC4581827

[33] Stoltzfus JC, Nishijima D, Melnikow J. Why quantile regression makes good sense for analyzing economic outcomes in medical research. Acad Emerg Med. 2012;19: 850–851. doi: 10.1111/j.1553-2712.2012.01386.x 22724505 10.1111/j.1553-2712.2012.01386.x

[34] Olsen CS, Clark AE, Thomas AM, Cook LJ. Comparing Least‐squares and Quantile Regression Approaches to Analyzing Median Hospital Charges. Acad Emerg Med. 2012;19: 866–875. doi: 10.1111/j.1553-2712.2012.01388.x 22805633 10.1111/j.1553-2712.2012.01388.x

[35] Fitzenberger B, Wilke RA. Quantile Regression Methods. Emerging Trends in the Social and Behavioral Sciences: An Interdisciplinary, Searchable, and Linkable Resource. 2015: 1–18.

[36] Madadizadeh F, Asar ME, Bahrampour A. Quantile Regression and its Key Role in Promoting Medical Research. Iranian journal of public health. 2016;45: 116 27057535 PMC4822385

[37] Lo T, Parkinson L, Cunich M, Byles J. Factors associated with higher healthcare costs in individuals living with arthritis: evidence from the quantile regression approach. Expert review of pharmacoeconomics & outcomes research. 2015;15: 833–841. 25896664 10.1586/14737167.2015.1037833

[38] Zhang JH, Chou SY, Deily ME, Lien HM. Hospital ownership and drug utilization under a global budget: a quantile regression analysis. Int Health. 2014;6: 62–69. doi: 10.1093/inthealth/ihu001 24526003 10.1093/inthealth/ihu001

[39] Frölich M, Melly B. Estimation of quantile treatment effects with Stata. The Stata Journal. 2010;10: 423–457.

[40] Gould W. Quantile regression with bootstrapped standard errors. Stata Technical Bulletin. 1993;2.

[41] Rogers W. Quantile regression standard errors. Stata Technical Bulletin. 1993;2.

[42] Wong LP, AbuBakar S, Chinna K. Community knowledge, health beliefs, practices and experiences related to dengue fever and its association with IgG seropositivity. 2014 doi: 10.1371/journal.pntd.0002789 24853259 10.1371/journal.pntd.0002789 PMC4031145

[43] Wong LP, Shakir SM, Atefi N, AbuBakar S. Factors affecting dengue prevention practices: nationwide survey of the Malaysian public. PLoS One. 2015;10: e0122890 doi: 10.1371/journal.pone.0122890 25836366 10.1371/journal.pone.0122890 PMC4383514

[44] Verbrugge LM. Gender and health: an update on hypotheses and evidence. J Health Soc Behav. 1985: 156–182. 3905939

[45] Rosenstock IM. Why people use health services. Milbank Q. 2005;83: Online‐only-Online‐only.

[46] Cao P, Wang K, Zhang H, Zhao R, Li C. Factors influencing the hospitalization costs of patients with type 2 diabetes. Asia-Pacific Journal of Public Health. 2015;27: 55S–60S. doi: 10.1177/1010539515573831 25834270 10.1177/1010539515573831

[47] Stahl HC, Butenschoen VM, Tran HT, Gozzer E, Skewes R, Mahendradhata Y, et al Cost of dengue outbreaks: literature review and country case studies. BMC Public Health. 2013;13: 1048-2458-13-1048. 10.1186/1471-2458-13-1048 PMC4228321 24195519

[48] Yoshioka R, Yasunaga H, Hasegawa K, Horiguchi H, Fushimi K, Aoki T, et al Impact of hospital volume on hospital mortality, length of stay and total costs after pancreaticoduodenectomy. Br J Surg. 2014;101: 523–529. doi: 10.1002/bjs.9420 24615349 10.1002/bjs.9420

[49] Otake H, Yasunaga H, Horiguchi H, Matsutani N, Matsuda S, Ohe K. Impact of hospital volume on chest tube duration, length of stay, and mortality after lobectomy. Ann Thorac Surg. 2011;92: 1069–1074. doi: 10.1016/j.athoracsur.2011.04.087 21871302 10.1016/j.athoracsur.2011.04.087

[50] Zhang Q, Lauderdale D, Mou S, Parish WI, Laumann EO, Schneider J. Socioeconomic disparity in healthcare-seeking behavior among Chinese women with genitourinary symptoms. Journal of Women's Health. 2009;18: 1833–1839. doi: 10.1089/jwh.2009.1394 19951219 10.1089/jwh.2009.1394 PMC2828239

[51] Lin Y, Zhang Q, Chen W, Shi J, Han S, Song X, et al Association between social integration and health among internal migrants in ZhongShan, China. PloS one. 2016;11: e0148397 doi: 10.1371/journal.pone.0148397 26863008 10.1371/journal.pone.0148397 PMC4749174

[52] Hagenlocher M, Delmelle E, Casas I, Kienberger S. Assessing socioeconomic vulnerability to dengue fever in Cali, Colombia: statistical vs expert-based modeling. Int J Health Geogr. 2013;12: 101186. doi: 10.1186/1476-072X-12-10 23945265 10.1186/1476-072X-12-36 PMC3765508

